# Considerations on imaging the PM/AICD patient; are we entering a new era? An evolutionary report of our first 100 Patients

**DOI:** 10.1186/1532-429X-17-S1-P240

**Published:** 2015-02-03

**Authors:** Robert W Biederman, June A Yamrozik, Ronald B Williams, Geetha Rayarao, Diane V Thompson, Huma Samar, Moneal Shah, Mark Doyle

**Affiliations:** Cardiac MRI, Allegheny General Hospital, Pittsburgh, PA USA; Cardiology, VA Medical Center, Los Angeles, CA USA

## Background

Pacemaker/AICD use may no longer be prohibitive in the MRI environment. Seminal work by us and others has pointed towards increasing safety and specifically, the marked additive clinical value. Historically, only extraordinarily high-risk patients with acute life-threatening diagnoses were imaged. However, over time, we began to note an interesting trend as our understanding, science and comfort level admixed with zero-event rate almost imperceptibly causes us to ‘relax' the mandate for ‘acuity'. We wondered, "Has imaging a patient with a pacemaker that was once considered a last resort procedure started to evolve into a ‘routine' study?

### Hypothesis

We hypothesize that there may be a learning curve with CMR imaging patients with PM/AICD's?

## Methods

We tracked the indication and matched them to date and type of CMR exam performed grouped into annual increments of our PM/AICD' over 10 consecutive years. Unchanged commonalities included scanner (GE 1.5T, Milwaukee, WI), 2 technologists and the CMR MD. ‘Acute' meaning life-endangering diagnosis/presentation was established by agreement between the scanning technologist and the MD. A total of 100 patients were imaged on a GE CV/i Excite Version 12, 1.5 T CRM (GE, Milwaukee, WI). In total, 62 patients had a complete pacemaker implantation, 17 patients had an AICD, 10 patients had an PM/AICD, 5 patients had a single PM lead and8 REVO PM's (1 not approved for Cardiac pacemaker imaging). The cardiologist had the final word after conversing with the ordering physician if the patient warranted this procedure. All patients signed informed consent.

## Results

**Figure 1:**
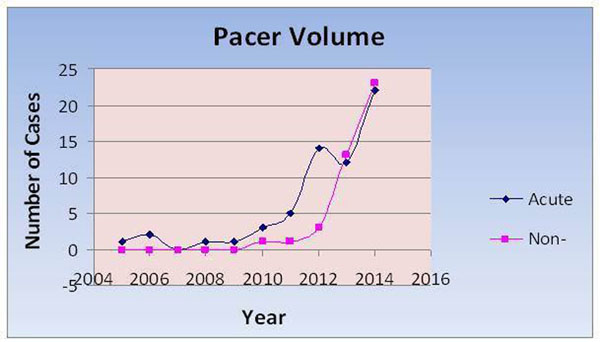
**Examining the temporal acuity relationship, we observed a similar relationship.** Namely, with time, the indication innocuously expanded such that proportionately, non-life threatening cases (cervical, lumbar, viability, cardiac function and knee) comprised an increasing population (p<0.05).

A total of 100 patients with implanted PM, AICD's or combination underwent scanning. During the early stages (2005-2011), a total of 15 all acute patients completed exam with no adverse events and all with cute need for a CMR. In 2012: 17pts, 2013: 25pts, and in 2014: 43pts underwent scanning again without incident. In 2014, the number almost doubled to 43 patients and in some instances was considered the first option to derive the optimal diagnosis (Fig 1).

## Conclusions

In what was once considered a last resort CMR exam is now becoming a more routine event. Confidence in performing CMR-PM/AICD imaging along with a zero adverse event rate has led our Specialized-CMR Center towards expanded indications for patients potentially candidates for ‘high-risk' imaging. We conclude that no longer is the bar set so high that only patients with acute diagnoses are eligible for CMR but rather non-acute, more routine diagnoses are being imaged suggesting that there *is* a learning curve.

## Funding

Internal.

